# Neural correlates of adversity-overcoming pup rescue behavior in female mice

**DOI:** 10.1038/s41598-026-35639-7

**Published:** 2026-03-03

**Authors:** Kseniia Prokofeva, Mizuki Shibamiya, Rin Kawata, Chihiro Yoshihara, Kumi O. Kuroda

**Affiliations:** 1 School of Life Science and Technology, Institute of Science Tokyo, Yokohama, 226-8501 Kanagawa Japan; 2 Laboratory for Circuit and Behavioral Physiology, RIKEN Center for Brain Science, Wako, 351-0198 Saitama Japan

**Keywords:** Alloparental care, Pup retrieval, Rescue, C-Fos, Altruism, Evolution, Neuroscience, Zoology

## Abstract

Rescuing infants under threat is a fundamental parental behavior in mammals. However, the behavioral expression and neural correlates of adversity-overcoming infant rescue in non-parental female individuals remain poorly understood. In this study, we first established a novel pup rescue paradigm with scalable adversity, in which mothers and virgin female mice have to cross a water pool of varying depths (0, 3, or 20 mm) to retrieve pups into the nest. We unexpectedly found that virgin females were less averse to water and retrieved pups faster than mothers. Next, we implemented an additional hurdle by trapping pups into a tube, so that female mice had to cross the pool and open the tubes to rescue pups. The rescuer virgin females in this “trapped pup” rescue task showed increased neuronal activity in the anterior cingulate cortex, lateral septum, anterior commissural nucleus, basolateral amygdala, and dorsal raphe nucleus, compared with non-rescuers. The c-Fos^+^ cell densities in these regions showed significant negative correlations with the latencies to rescue behaviors suggesting their positive impact on rescue. Given that the virgin females do not have genetic relations to the rescuee pups, our findings provide a basis for further analyses of adversity-overcoming altruistic behavior and its neural correlates.

**Supplementary Information:**

The online version contains supplementary material available at 10.1038/s41598-026-35639-7.

## Introduction

Targeted helping-like behaviors toward conspecific adults, expressed as freeing them from restraint or adverse environments or as allogrooming stressed conspecifics, have been observed in several rodent species under laboratory conditions^1–8^. Recent studies have also reported that mice engage in resuscitation-like behaviors toward unresponsive conspecifics via methodical interactions with the tongue and head of the rescuees^9–11^. However, it has been questioned whether such behaviors in rodents arise for the other-helping purpose or from selfish intentions, such as the avoidance of being alarmed or as preparation for a potential threat^12,13^. “Altruism” has been defined as the behavior that benefits the receiver at a cost (loss) to the performer^14,15^. Therefore, to investigate the altruistic nature of helping-like behavior, an ideal behavioral paradigm should impose a significant, objectively measurable environmental cost, which is avoided, on the performer, however, such studies are limited^8,16–18^.

Infant care is a paramount mammalian behavior that facilitates the survival of the young, and it requires a substantial cost from the caregiver^19,20^. Previous studies including ours have shown that rodent mothers take significant risks to rescue their pups, for example, by attacking intruder males or by bringing pups placed at the ends of an open elevated platform to a safe closed arm (pup retrieval), while virgin females do so less^21–24^. We have previously shown that the risk-taking pup rescue from the open arms of an elevated plus maze in mice requires specific gene expression in the anterior commissural nucleus (ACN) and the caudocentral part (cMPOA) of the medial preoptic area (MPOA), which is an indispensable hub for infant care motivation in mammals^20,25^.

The performance of the risk-taking pup rescue, however, must be influenced not only by the motivation to care but also by risk assessment and cognitive/behavioral skills to cope with the specific risk. Thus, the neural circuit for such a task should involve not only the MPOA but also other brain regions. To elucidate the whole neural circuit required for such an adversity-overcoming pup rescue, it is preferable to utilize a behavioral paradigm with progressively increasing, gradating environmental risk where the risk assessment can be reliably examined in each animal. However, it was not easy in our previous pup rescue paradigm on the elevated plus maze.

To address this issue in this study, we established an adversity-overcoming pup rescue task that involves crossing a water pool of variable depths to retrieve (rescue) pups, utilizing the previous findings that mice are averse to entering water^26–29^. We tested pup rescue behaviors of mothers and virgin females in this behavioral task and surprisingly found that virgins rescue the pups placed behind the water pool better than mothers. Therefore, we screened for the brain areas that are activated during this behavioral task in virgin females, using immunohistochemistry and c-Fos analysis. As we utilized female virgin mice that are not kin to the rescuee pups, our histological study sheds light not only on the neural correlates of costly pup rescue, but also on those of the altruistic helping-like behavior.

## Results

### Mice perceive increasing water depth levels as scalable adversity

**Fig. 1:**
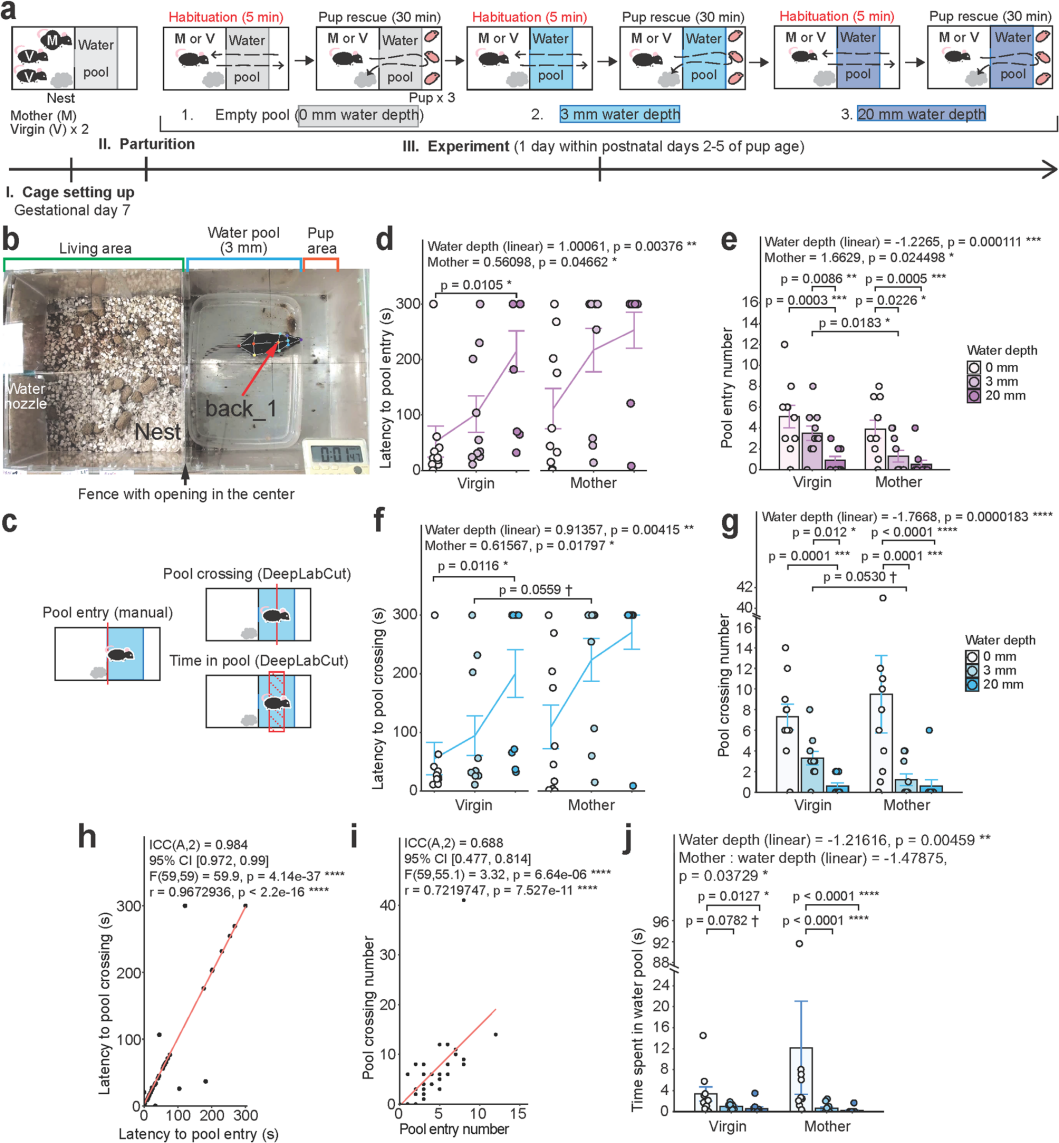
Examination of aversion to water in virgin females and mothers and establishment of automatic video analysis. **a**, Scheme of the experiment and order of trials (adversity levels). Red font denotes the part of the experiment (habituation) analyzed in Fig. 1. **b**, Representative image of the experimental environment and utilized DeepLabCut (DLC) model. Pup area denotes the location where pups will be placed during the pup rescue test (see Supplementary Video S1). **c**, Visualization of manually (left panel) and automatically (right upper and bottom panels) measured parameters in Fig. 1. **d**, Latency to the first pool entry in seconds (generalized linear mixed model (GLMM), latency ~ mouse type * water depth + (1 | mouse ID), gamma distribution). Water depth independent variable was set as ordinal and GLMM was followed by Holm (between mouse groups) or Tukey (between water depth trials) multiple comparisons in this and following models. Effect size is reported in log scale in this and following models, unless stated otherwise. **e**, Number of water pool entries (GLMM, latency ~ mouse type * water depth + (1 | mouse ID), negative binomial distribution). **f**, Latency to the first pool crossing in seconds (GLMM, latency ~ mouse type * water depth + (1 | mouse ID), gamma distribution). **g**, Number of pool crossings (GLMM, latency ~ mouse type * water depth + (1 | mouse ID), negative binomial distribution). **h**, Correlation plot between latencies to the first pool entry and the first pool crossing (Pearson correlation). Red line represents linear regression. **i**, Correlation plot between numbers of pool entries and pool crossings (Pearson correlation). **j**, Time spent in the water pool (GLMM, latency ~ mouse type * water depth + (1 | mouse ID), tweedie distribution). Data are shown as mean ± S.E.M. Virgin mice, n = 10. Mother mice, n = 10.

**Fig. 2:**
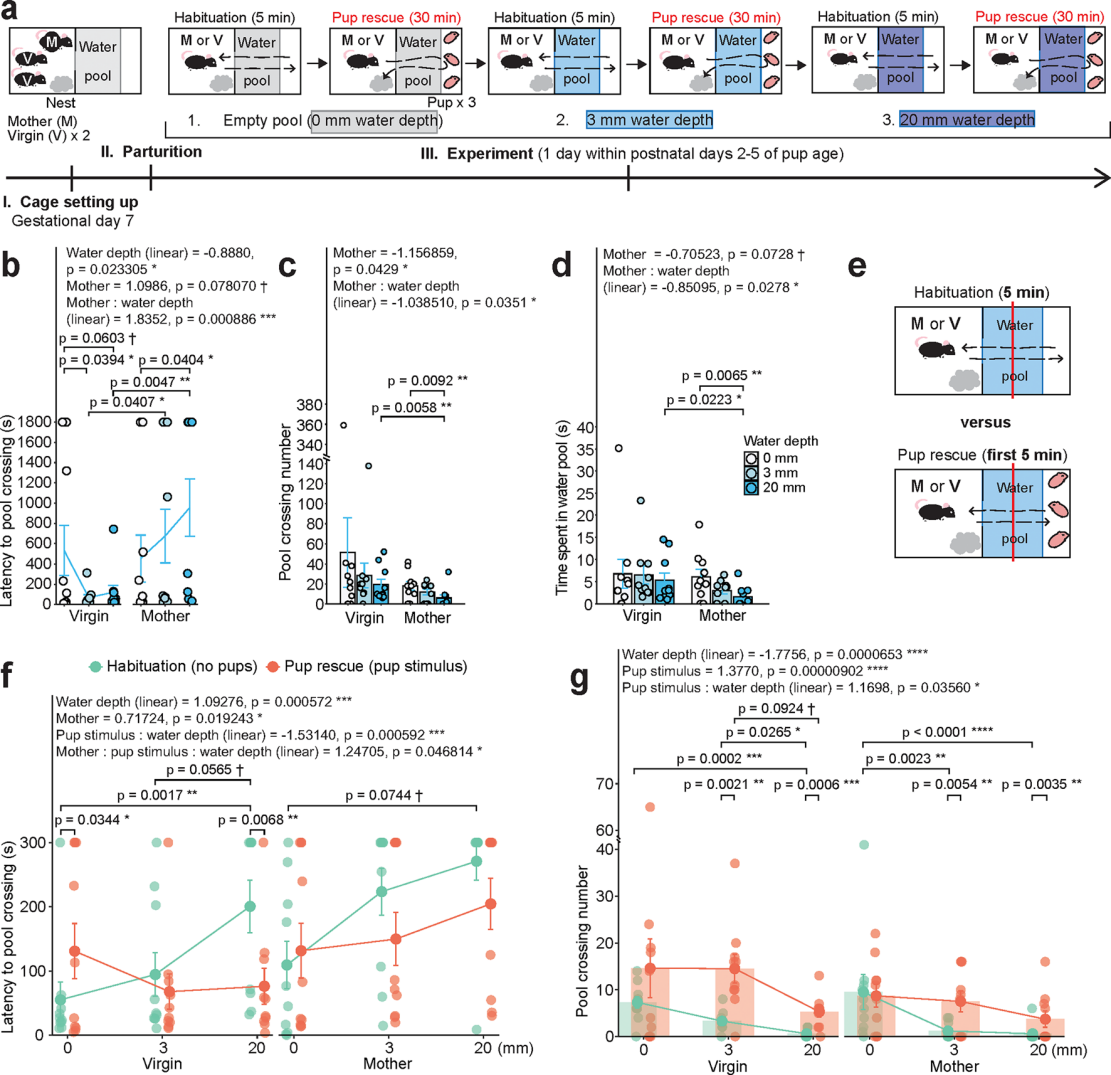
Analysis of adversity-overcoming behavior during pup rescue in virgin females and mothers. **a**, Scheme of the experiment and order of trials. Red font denotes the part of the experiment (pup rescue) analyzed in Fig. 2. **b**, Latency to the first pool crossing in seconds (GLMM, latency ~ mouse type * water depth + (1 | mouse ID), gamma distribution). **c**, Number of pool crossings in seconds (GLMM, latency ~ mouse type * water depth + (1 | mouse ID), negative binomial distribution). **d**, Time spent in the water pool (GLMM, latency ~ mouse type * water depth, tweedie distribution). **e**, Scheme of the analysis concept for Figs. 2f, g. **f**, Latency to the first pool crossing in seconds during first 5 min of habituation and pup rescue tests (GLMM, latency ~ test type * mouse type * water depth + (1 | mouse ID), gamma distribution). **g**, Number of pool crossings during first 5 min of habituation and pup rescue tests (GLMM, latency ~ test type * mouse type * water depth + (1 | mouse ID), negative binomial distribution). Data are shown as mean ± S.E.M. Virgin mice, *n* = 10. Mother mice, *n* = 10.

**Fig. 3:**
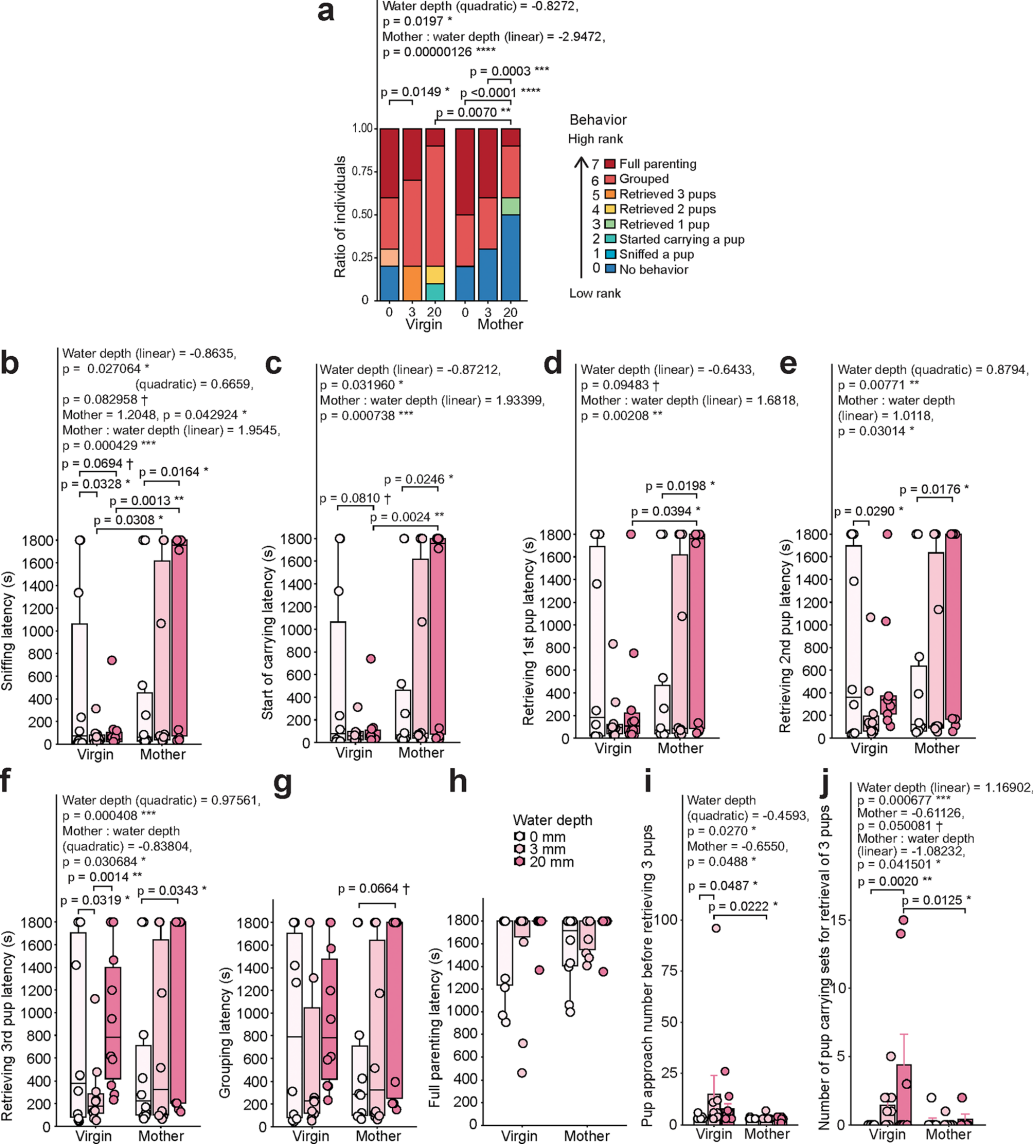
Analysis of pup care behaviors in adversity-overcoming rescue paradigm in virgin females and mothers. **a**, Stacked bar graph displaying ratio of mice (virgins and mothers) that performed or did not perform pup-directed behaviors at 0 mm, 3 mm, or 20 mm water depth levels. First, each analyzed behavior was assigned a number according to its rank (e.g., 7 – full parenting, 0 – no behavior). Next, for each individual mouse, the highest achieved behavior was identified and set as the corresponding number. These numbers were then compared. The distribution was set to “binomial” (due to the presence of ceiling value of 7), and water depth independent variable was set as ordinal (GLMM, cbind(value, 7 - value) ~ mouse type * water depth + (1 | mouse ID)). Effect size is reported in logit scale. **b-h**, Latencies to the pup sniffing (**b**), start of pup carrying (**c**), first pup retrieval (**d**), second pup retrieval (**e**), third pup retrieval (**f**), pup grouping (**g;** GLMM, latency ~ mouse type * water depth + (1 | mouse ID), gamma distribution), and full parenting (**h**) in seconds. Due to rarity of full parenting behavior, statistical analysis was not applied to it. **i**, Number of pup approaches before retrieval (sum for three pups; GLMM; latency ~ mouse type * water depth, negative binomial distribution). 0 mm: Virgins, *n* = 10; Mothers, *n* = 8. 3 mm: Virgins, *n* = 10; Mothers, *n* = 7. 20 mm: Virgins, *n* = 6; Mothers, *n* = 5. **j**, Number of pup carrying sets for pup retrieval from original position into the nest (sum for three pups; GLMM; latency ~ mouse type * water depth, negative binomial distribution). 0 mm: Virgins, *n* = 9; Mothers, *n* = 8. 3 mm: Virgins, *n* = 9; Mothers, *n* = 7. 20 mm: Virgins, *n* = 6; Mothers, *n* = 5. If mouse retrieved only one or two pups, the number of approaches and sets were divided by the total number of pups (3) and multiplied by the number of retrieved pups. Data are shown as mean ± S.E.M. Virgin mice, *n* = 10. Mother mice, *n* = 10, unless noted otherwise.

We have implemented an adversity-overcoming pup rescue paradigm using mothers and pup care-experienced virgin females that have been cohoused from the gestational day 7 of each mother (Fig. 1a; Supplementary Video S1). After delivery, both mothers and virgins were separately subjected to three consecutive pup rescue tests under three increasing water depths levels, which were conducted in one day for each mouse within the period of postpartum days 2–5. Before each pup rescue test described in later Figs. 2 and 3, we habituated subject mice to each water pool depth (0 mm = no water, 3 mm, and 20 mm) for 5 min (this period is termed “habituation”) and examined the subjects’ interaction with the water pool (Fig. 1a, b).

First, we manually measured the latency to the first pool entry and the number of pool entries (Fig. 1c, left panel). As the water depth increased, both the mother and the virgin females required significantly longer time to enter the pool and entered the pool significantly less (Fig. 1d, e; GLMM; see Supplementary Table S1). Virgins entered the empty pool significantly faster and more than the 20 mm-deep pool, while there was a significant decrease in the pool entry number between the 3 mm- and 20 mm-deep water conditions (Fig. 1d, e; GLMM followed by Tukey *post-hoc* test). Mothers entered the pool significantly less in both water conditions compared with the empty pool (Fig. 1e; GLMM followed by Tukey *post-hoc* test). Notably, virgins entered the pool significantly faster and more often than mothers with particularly higher entry numbers in the 3 mm water condition (Fig. 1d, e; GLMM followed by Holm *post-hoc* test), suggesting that water was recognized by mothers as stronger adversity. Altogether, we confirmed that mice are increasingly averse to water upon the rise in water depth, while maternity status exacerbates the perception of adversity.

To optimize the analyses of behavioral parameters, we implemented a machine learning-based animal motion tracking program DeepLabCut (DLC)^30^ to measure the latency to the first pool crossing and the number of pool crossings, which roughly correspond to pool entry parameters, utilizing the middle point of mouse body to analyze its locomotion (Fig. 1b; back_1; Fig. 1c, right panels). We found that both latency to pool crossing and the pool crossing number followed a similar trend to the pool entry parameters using both GLMM and Cox regression (for latency only) (Fig. 1f, g; GLMM followed by Holm or Tukey *post-hoc* tests; see Supplementary Table S1; Supplementary Figs. S1a, b).

The automated measures of the latencies to and the number of pool crossings were significantly correlated with the manual measures (Figs. 1h, i), suggesting that manual and DLC analyses are essentially interchangeable. We additionally conducted intraclass correlation (ICC) analyses between the manual and DLC methods and found that they yielded high similarity (Fig. 1h, i). Using DLC, we also analyzed time spent in the water pool, which aligned with other parameters and confirmed mouse aversion to water (Fig. 1j). We therefore established a DLC-mediated automatic analysis pipeline and used it for further behavior examination.

### Female virgins overcome adversity to rescue pups

Next, we conducted a pup rescue test and examined pup-directed behaviors performed by mothers and virgin females (Fig. 2a). When mouse pups are placed outside of the nest, female mice generally quickly recognize them by sniffing and engage in a sequence of behaviors, starting from orally carrying the pup, placing the pup into the nest (retrieving), gathering pups within the nest (grouping), and crouching over pups (designated as full parenting = crouching over all pups continuously for over a minute), as reported before^19,25,31^. In the pup rescue test, we examined these behaviors toward three pups of the postnatal age 2–5 days placed behind the water pool and designated the act of adversity-overcoming pup retrieval as pup rescue.

We first investigated the level of water aversion during the pup rescue test using the latency to the first pool crossing (Fig. 2b), the number of pool crossings (Fig. 2c), and the time spent in the pool (Fig. 2d; GLMM followed by Holm or Tukey *post-hoc* tests, see Supplementary Table S2). Overall, as in habituation tests, mothers tended to show stronger water aversion than virgins (Fig. 2b-d). Mothers also displayed the water depth-dependent pool aversion in pup presence. In contrast, the depth-depended water aversion appeared to be absent in virgin females in presence of pups (Fig. 2b-d), and the mean latency to pool crossing was longest in the 0 mm trial. It could have been a stochastic effect, as the number of pool crossings and time spent in the pool failed to confirm virgins’ avoidance of the empty pool (Figs. 2c, d).

We next directly compared pool crossing behavior in the absence (habituation) and presence of pups behind the water pool (the first 5 min of the pup rescue test; Fig. 2e-g). We found that mice generally avoided deep water and that pup presence significantly reduced the latency to the first pool crossing and increased the number of pool crossings as the water depth increased (Figs. 2f, g; GLMM followed by Tukey *post-hoc* test). These results suggest that pool crossing behavior was influenced by both aversion to the water and motivation for pup care (“net motivation” = motivation for pups - adversity perception). Notably, the latency to pool crossing was larger in mothers compared with virgins in the pup presence and as the water depth increased, suggesting that for mothers the water was still a stronger adversity than for virgins (Fig. 2f).

We next focused on pup-directed behaviors and ranked them based on their achievement (e.g., no pup-directed behavior – 0 (lowest rank), pup sniffing – 1, and full parenting, which is only possible if all other behaviors were performed, – 7 (highest rank)). We found that a large proportion of female virgins and mothers were able to retrieve and nurture pups at any adversity level. However, while performing similarly to mothers in the very first trial with no water in the pool, virgins unexpectedly displayed better pup retrieval behaviors as adversity increased, causing the non-linear correlation between the water depth and the behavioral performance (Fig. 3a; GLMM followed by Holm or Tukey *post-hoc* tests). Mothers, on the other hand, showed linear decrease of pup care along the water depth increase and performed significantly worse than virgins in the 20 mm trial. These results were in accordance with the pool crossing behavior in both virgins and mothers (Fig. 2b–d), suggesting that the observed behavioral dynamics of pup care were strongly tied to the environmental and adversity factors. Altogether, we found that female virgins are more likely to overcome adversity and display nurturing behaviors than mothers.

We then identified the latencies to each pup-directed behavior to examine motivation more precisely (Fig. 3b-h; GLMM followed by Holm or Tukey *post-hoc* tests; see Supplementary Table S3). We found that virgins sniffed pups upon crossing the water pool significantly faster than mothers (Fig. 3b), while the latencies to both sniffing and first pup carrying were increased in both mouse groups with an increase in water depth (Figs. 3b, c). Additionally, similarly to our earlier observations, latencies to pup sniffing, carrying, and retrieving the first two pups lengthened with the increase of adversity in mothers more (Figs. 3b-e). Similarly to previous findings (Fig. 3a), the latencies to retrieve the second and third pup were stabilized upon the addition of water to the pool in all mice (Figs. 3e, f), however, this effect was not as present in mothers as in virgins (Fig. 3e). We did not observe any differences in grouping behavior and did not conduct statistics on full parenting due to its rarity (Figs. 3g, h). Additionally, as mice often could not perform observed behaviors, we analyzed all latency data using Cox regression and found that the results were largely aligned with the above ones, however, Cox regression failed to observe linear relationships of latencies with water depth increase (Supplementary Fig. S1c-j, see Supplementary Table S4). Among virgins, they engaged in the first pup sniffing, as well as in retrieving the second and third pups, significantly faster in the 3 mm trial than in the first trial (Figs. 3b, e, f; GLMM followed by Tukey *post-hoc* test), consistent with the previous data (Fig. 2b). Mothers routinely took longer time to sniff, carry, and retrieve each pup in the 20 mm water depth trial compared with no water trial (Fig. 3b-f; GLMM followed by Tukey *post-hoc* test).

During this study, we noted that mothers displayed better pup care skill than virgin females, despite being significantly hindered by the adversity. To quantify the skill difference, we counted the total number of pup approaches before engaging in retrieval of all three pups and the number of carrying sets needed to successfully retrieve three pups both in virgins and mothers (Figs. 3i, j). Generally, mothers approached pups fewer times before retrieving, while the number of approaches in each trial followed non-linear relationship with water depth (Fig. 3i; GLMM followed by Holm or Tukey *post-hoc* tests). Mothers also showed a trend of doing fewer carrying sets to retrieve pups (Fig. 3j; GLMM followed by Holm or Tukey *post-hoc* tests). The number of sets generally increased with water depth increase, but at the same time decreased more in mothers. These results suggest that, while mothers retrieved pups in minimal carrying bouts, virgins were less skilled and often dropped pups, especially when the pool had more water. However, the results must be interpreted cautiously due to the differing number of successfully retrieving animals both between mouse groups and between trials (see corresponding figure legends). Therefore, we concluded that virgins are more resilient to water adversity in pup presence and rescue pups better than mothers, even though they are less skilled at doing so.

### Costly “trapped pup” rescue increases activity in empathy-, infant care-, and adversity-associated brain areas

**Fig. 4:**
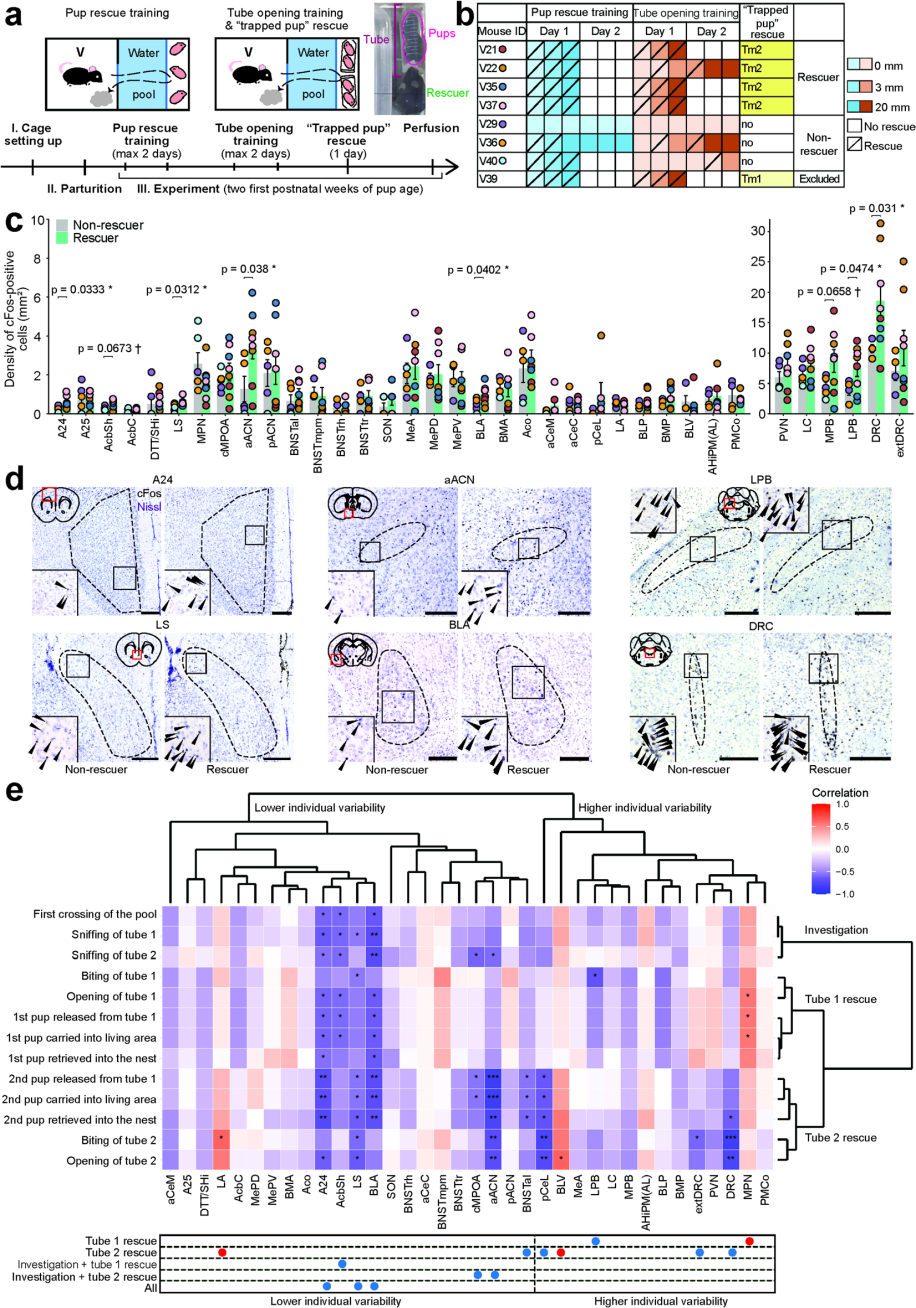
Brain regions activated during adversity-overcoming “trapped pup” rescue in virgin females. **a**, Scheme of the experiment followed by brain sampling for c-Fos examination and representative image of “trapped pups” rescue. *V* virgin female. **b**, Behavior achievement during training and “trapped pup” rescue on each day by each mouse. *Tm2* opening both tubes, *Tm1* opening the first tube only. Colors next to mouse ID represent color code for Fig. 4c. **c**, Density of c-Fos-positive cells in rescuers and non-rescuers in each examined brain region (independent Welch’s t-test for each brain region, unadjusted p-values). Adjusted p-values and replicate number for each region are reported in the Supplementary Table S5. Data are shown as mean ± S.E.M. Rescuer, n = 5-8, non-rescuer, n = 4-6 (including technical replicates of the left and right brain hemispheres). We obtained four brain samples from the first group and three samples from the second group. Colors represent mouse ID. **d**, Representative images of brain regions with significantly higher c-Fos density in rescuers based on independent Welch’s t-tests. Scale bar: 500 μm. Black arrows show c-Fos-positive cell nuclei. **e**, A heatmap demonstrating Spearman correlation results between c-Fos^+^ cell densities in various brain regions and latencies to measured behaviors in the “trapped pup” rescue paradigm. Panels below the heatmap mark brain regions that have significant correlation coefficients with different behavioral clusters where the blue color corresponds to negative correlation and red corresponds to positive correlation. Spearman correlation coefficients, unadjusted and adjusted p-values are reported in Supplementary Table S7.

To identify brain regions implicated in adversity-overcoming pup rescue, we examined the expression of the cellular activity marker c-Fos across the brain during this behavior. For this purpose, we made two minor changes to the experimental protocol. First, we restricted the spontaneous locomotion of the stimulus pups by containing them in a plastic tube (two pups per tube) with a paper lid (“trapped pup”)^2,32^. Second, before the final pup exposure test, we trained the subject females for a period of up to four days, so that the subjects could successfully rescue pups even under adverse conditions and when pups were contained in a plastic tube (Fig. 4a; Supplementary Video S2). In the first phase of training, the subjects were directly exposed to three pups (i.e., without being contained in a tube as in Fig. 3) placed on the opposite side of the pool with the test being repeated at each water depth level until the subjects succeeded or until the maximum length of the training period (two days) had been reached. In the second phase of training (tube opening), we placed two 50-ml tubes each containing two pups behind the pool and repeated the test in a similar manner, while we counted rescue success as tube opening. After the training, the subjects were isolated for one day and then underwent the final experiment (“trapped pup” rescue) similarly to the last phase of training, but only at the 3 mm water depth. Mice that opened both tubes within the first 30 min were designated as “rescuers”. Out of four “rescuer” animals, most of them (3/4, 75%) opened the first tube and retrieved at least one pup before opening the second tube, while one (25%) first opened both tubes and then retrieved the pups. Notably, none of the animals opened the tubes without retrieving the pups. These results suggest that mice exhibit behaviors in an organized manner with the aim of pup rescue, rather than out of interest in the tubes. Mice that did not open any of the tubes during the task displayed a range of behaviors, from complete lack of interest in the pool and pup-containing tubes to active pool crossing and tube sniffing without biting. Thus, these mice were designated as “non-rescuers” (control group), while mice with intermediate performance were excluded from the c-Fos analysis. Following this behavioral experiment, mice were subjected to brain sampling (Fig. 4b).

We next compared c-Fos expression across the brain between the rescuers and non-rescuers using the data from both hemispheres of each subject (technical replicates) for analysis (Fig. 4c, d; independent Welch’s t-test for each brain region; see Supplementary Table S5). We selected the target brain regions involved in pup nurturing, emotion processing, and empathic response^5,8,9,11,13,16,17,31,33–42^. The “rescuer” group exhibited a significantly higher density of c-Fos-positive cells in the anterior part of the anterior commissural nucleus of the preoptic area (aACN), which has already been shown to be activated most after parental nurturing behaviors^33^. Three limbic regions, that are the area 24 of the anterior cingulate cortex (A24), lateral septum (LS), and basolateral amygdala (BLA), were also activated in rescuers. Finally, several hindbrain regions, such as the caudal part of the dorsal raphe nucleus (DRC) and the lateral parabrachial nucleus (LPB), displayed higher c-Fos cell density in rescuers compared with non-rescuers. The shell of the nucleus accumbens (AcbSh) and medial parabrachial nucleus (MPB) showed tendencies (0.05 < *p* < 0.1) toward an increase of the density of c-Fos-positive cells in the rescuer mice. The locus coeruleus (LC) and paraventricular nucleus (PVN), which are known to be activated by stress, exhibited relatively high c-Fos densities in both groups. No brain region in the Fig. 4c showed higher activity in the non-rescuer group. To account for using technical replicates, we also conducted LMM treating hemisphere data as repeated measures for each subject (LMM, density ~ hemisphere * brain area + (1|mouse.ID), gaussian distribution, followed by FDR multiple comparisons test). The analysis revealed a significant main effect of brain region (F₃₅,₃₆₉.₄₉ = 24.06, *p* < 0.001) and a significant mouse group and region interaction (F₃₅,₃₆₉.₄₉ = 1.75, *p* = 0.0067), indicating that group differences varied across regions. The main effect of mouse group was not significant (F₁,₅.₁₈ = 3.40, *p* = 0.12). Multiple comparisons showed that MPB and DRC had significantly increased c-Fos cell densities in rescuers compared with non-rescuers, while the area surrounding DRC (extDRC) and LPB displayed a trend toward increase (Supplementary Table S6; LMM followed by FDR *post-hoc* test), highlighting the strong activation of these regions in response to pup rescue in addition to previous results (Fig. 4c).

### Identification of neural correlates of adversity-overcoming “trapped pup” rescue

To better describe the link between the behavior and rescue-biased brain regions, we next analyzed correlations between the latencies to the major components of rescue behavior, such as crossing the pool and biting each tube, and within-region standardized densities of c-Fos-positive cells in the analyzed brain regions (Fig. 4e; Spearman correlation; see Supplementary Table S7). The behaviors and activation patterns of the brain regions were each sorted using unsupervised hierarchical clustering where the clusters were formed based on similarity between items.

The behavior clustering aligned with the chronological sequence of displayed behaviors: investigation of the pups, opening the first tube followed by retrieval of the first pup from it (tube 1 rescue), and retrieving the second pup from the first tube followed by opening the second tube (tube 2 rescue). The clustering of brain areas was based on c-Fos density variability between individual mice, highlighting the consistency of each area’s response to the examined behaviors.

The correlation coefficients between the brain region activities and the latencies to each behavior are color-coded in Fig. 4e. Negative correlation (blue) means that the given brain region was more active when mice performed the given behavior at a short latency. The activities of the aACN and cMPOA were negatively correlated with the latencies to the chronologically later behaviors, particularly with the latencies to the tube 2 rescue, implicating these brain regions in high rescue motivation. The four limbic areas, A24, LS, BLA, and AcbSh, that showed a (trend of) higher activation in the rescuer group, were clustered together and exhibited negative correlations with latencies to the behaviors in all chronological clusters, suggesting their involvement through negative emotions and affective states processing generally associated with our behavioral task. The DRC and LPB also showed chronologically graded correlation patterns yet were not classified together with the aACN and limbic regions due to higher inter-mouse variability of c-Fos density. The precise roles of these and other brain regions demonstrating significant correlations should be determined in future studies.

## Discussion

This study investigated adversity-overcoming pup rescue behavior in postpartum and virgin female mice, using a new behavioral paradigm based on mice’s known aversion to water^26–29^. Numerous studies in rodents showed that mothers care for pups better than female virgins, especially in difficult tasks or under stressful conditions^21,23,43,44^. Thus, we expected mouse mothers to outperform virgin females in our water pool-crossing pup rescue paradigm. Surprisingly, however, virgins clearly surpassed mothers in pup rescue (Fig. 3a), plausibly due to mothers’ heightened aversion to water compared with the virgin females’ one (Figs. 1d-g, j**)**, which retained even in pup presence. To our knowledge, such a strong aversion to a large body of water in postpartum mice has not been reported previously and might have occurred as a physiological adaptation to lactation (e.g., for energy conservation). Lactation-associated hormones oxytocin and prolactin are known to participate in thermoregulation^45,46^, but the precise governing mechanisms of heightened water aversion require further investigation. Our findings also suggest that the modality of environmental stress can be a critical determinant for parental care motivation in various reproductive contexts. For more comprehensive understanding of adversity-overcoming pup care, it may be beneficial to compare the results of experiments using different modalities of risks, such as water, bright light, or height, with different risk levels.

Several limitations of our behavioral paradigm should be considered. First, we conducted all trials in the set order of 0 mm, 3 mm, and 20 mm water depths, and therefore the influence of task experience on water aversion and rescue performance remains to be clarified. Second, we used only pup stimulus in the rescue experiments. As our behavioral paradigm allows the introduction and comparison of other social and non-social stimuli, especially if implementing the “trapped” paradigm, expanding stimulus types in future studies will help disentangle pup-specific motivation from ones for other social and general exploratory behaviors.

Because the rescuer virgin females in our “trapped pup” behavioral paradigm were not kin to the rescuee pups, we have approached this behavior (essentially, alloparental care) as prosocial and particularly altruistic^14,15^. We then explored the brain areas activated by the adversity-overcoming “trapped pup” rescue from the perspective of the known neural basis of prosocial behaviors^5,8–11,13,16,17,34,37–39,41,42^. Our findings at least partially aligned with the results of these studies, as we showed that A24, LS, BLA, DRC, and to a lesser extent AcbSh were activated during pup rescue, along with the preoptic area (aACN) crucial for parental care^33^ and several other regions (LPB, to a smaller degree MPB). However, we were unable to confirm an increased activity in the medial amygdala (MeA) and PVN, which might have been due to the differences in stimulus animals (pup versus adult) and their conscious awareness level, as well as related to environmental adversity and experienced stress. Identified brain regions may have been activated in response to pup cues and associated reward^25,36,47,48^ and/or as a reaction to an adverse and sensory-heavy environment^35,49^. Notably, absolute most of activated brain regions also showed strong relations with particular rescue behaviors, such as the tube biting or opening (Figs. 4c, e). Although demonstrating functional importance of identified brain regions is beyond the scope of this study, targeted manipulations of these areas during adversity-overcoming rescue will be an important next step.

Ethological and experimental evidence collectively suggest that alloparental care evolved from parental care, and that primate cooperative behaviors among adults may have derived from allomaternal care^20,25,50–53^. Although our study cannot reconstruct the evolutionary sequence, the shared activation of brain areas associated with parental and prosocial behaviors during pup rescue is consistent with these hypotheses. Examining the neural basis of adversity-overcoming rescue across pups of different ages and adult conspecifics could further illuminate the common mechanisms underlying alloparental care and altruism.

## Materials and methods

### Animals

All mouse experiments were approved by the Animal Care and Use Committee of the Institute of Science Tokyo and were thus in accordance with NIH guidelines (NIH Publications No. 8023, revised 1985). This study followed the ARRIVE Guidelines 2.0^54^. Animals were maintained under a 12:12 h light/dark cycle (7:00 lights on, 19:00 lights off) with food and water *ad libitum*. C57BL/6 N mice were originally obtained from Japan SLC or Jackson Laboratory and raised in our breeding colony. Pups were weaned at 4 weeks of age and housed with their littermates and/or mice of the same age in groups up to five. 2- to 10-months-old female mice were used for experiments (mean age of mothers 22.18571 weeks vs. mean age of virgins 21.74286 weeks; *p* = 0.849, Welch’s t-test).

### Adversity-overcoming pup rescue

A pregnant wild-type female mouse (gestational age 7~) was housed together with two virgin females in an experimental cage (44 × 24 × 15 cm, Fig. 1a) until delivery. The experimental cage included a living area, where a mouse nest was located, and an experimental area that was separated from the living area with a water pool and a sham metallic fence to force mice to enter the pool from the center. Virgin females underwent a pup retrieval test in their home cages in advance to confirm their nurturing behavior. Experiments, consisting of combined habituation and pup rescue tests at the same adversity level, repeated at three adversity levels per day in a fixed order, were conducted during the period of pup age of postnatal day 2–5 (PND2-5) between 9:30–13:00. The three levels of adversity and the order of trials were as follows: (1) 0 mm (empty pool), (2) 3 mm, and (3) 20 mm water depth in the pool in the experimental area, based on the results of a previous study^28^. The order of trials was fixed to follow the rule of implementing stress from low to high^55^. Room temperature water (temperature range between + 21.0 °C and + 23 °C) was used. The habituation and pup rescue tests were structured in the following way. One subject (a mother or a virgin) was left in the experimental cage, while other animals, including pups, were temporarily transferred to a clean husbandry cage. The subject was left in the empty cage with no water in the pool for 5 min (habituation test). After that, 3 healthy pups were placed behind the pool in the experimental area and the subject’s behavior was observed for 30 min. Next, the subject was habituated to the empty cage with 3 mm-deep water in the pool for 5 min, and then 3 healthy pups were placed behind the pool in a similar way to observe subject’s behavior for 30 min. Finally, the same tests were conducted with the 20 mm-deep water in the pool. Upon the end of these tests, all animals were reunited in the experimental cage. Next day, another subject underwent the experiment. 90% of experiments were conducted in the order of testing a mother on the first day and testing a virgin on the second day to decrease the effect of cage change stress on mothers’ performance and pup survival, while the rest of the experiments followed the opposite order. All experiments were recorded on video and analyzed manually and automatically using DeepLabCut^30^. Manual analysis was performed by several raters with exceptional inter-rater reliability (the absolute‑agreement, two‑way random‑effects Intraclass Correlation Coefficient (ICC) for k = 4 rater ICC(A,4) = 0.996 (95% CI 0.995–0.997; F(85, 257) = 262, *p* < 1 × 10⁻²¹⁰)).

### Adversity-overcoming “trapped pup” rescue for c-Fos sampling

To examine c-Fos activity during the pup rescue test, only virgin female mice were used. Two transparent empty 50 ml Falcon tubes were introduced into the experimental cage at least two times a week from the cage setup time until delivery to habituate mice to the tubes and make them learn the tube-opening behavior. Next, we started systematic training. First, we trained subjects to retrieve pups as described above at each water depth for one day (max three tests per day). If the subject failed to retrieve pups at a particular water depth, we started from this water depth the next day and repeated the tasks for one more day. Next, we conducted similar experiments but placed pups in two 50 ml Falcon tubes (2 pups/tube) closed with paper lids (“trapped pup”) to make pup rescue comparable with the one of adult conspecifics or older pups^1,2,37,38^ and repeated these tests for up to two days. After that, each subject was isolated in the husbandry cage overnight and then underwent the “trapped pup” rescue test at the 3 mm water depth in the pool. Mice were labeled as “rescuers” (= opened both tubes within 30 min) and “non-rescuers” (= did not open the tubes within 30 min), while subjects with intermediate performance were excluded from future studies. Two hours from the start of the test, mice were perfused, and brains were sampled. We did not use empty tubes or object-containing tubes as controls for observed behaviors in the final “trapped pup” rescue test because previous reports showed that mice can discriminate between mouse-containing and other tubes^2,32^.

### Brain tissue processing

Mice that underwent “trapped pup” rescue experiment were deeply anesthetized with 8% (v/v) Midazolam (Sandoz Group AG, Switzerland), 10% (v/v) Vetorphale (Meiji Animal Health Co., Ltd., Japan), 7.5% (v/v) Domitor (Nippon Zenyaku Kogyo Co., Ltd., Japan) in saline, then perfused transcardially with 4% (w/v) paraformaldehyde (PFA) in 1x phosphate buffered saline (PBS, pH 7.4). The brains were removed, immersed in the same fixative at 4 °C overnight, followed by cryoprotection in the series of 20% and 30% (w/v) sucrose in PBS for two days, embedded in O.C.T. Compound (Sakura Finetek Japan, Tokyo, Japan), and stored at -80 °C until cryosectioning. Brains were cryosectioned coronally at a thickness of 40 mm according to the mouse brain atlas^56^. Every third section from the serial sections was used for immunohistochemistry.

### Immunohistochemistry (IHC)

Immunohistochemical detection of c-Fos on free-floating sections was performed as described previously^33^. The sections were washed with PBS containing 0.2% Triton-100 (PBST), incubated with 0.3% H_2_O_2_ in methanol for 5 minutes, washed with PBST, blocked with 0.8% Block Ace (Dainihon-Seiyaku, Osaka, Japan) in PBST, and incubated at 4°C overnight with rabbit primary antibody against c-Fos (1:5000, Cat# sc-52, RRID: AB_2106783, Santa Cruz Biotechnology, Inc., Dallas, TX, USA). The following morning, the sections were washed and incubated with biotin-conjugated horse anti-rabbit secondary antibody (1:2000, Cat# BA-1100, RRID: AB_2336201, Vector Laboratories, Inc., Burlingame, CA, USA) for 2 hours and then in ABC peroxidase reagent (Cat# PK-6100; Vectastain ABC Elite kit; Vector Laboratories) for 1 hour according to the manufacturer’s instructions. The labeling was visualized by incubation in 3,30’–diaminobenzidine (DAB) solution with nickel intensification (DAB peroxidase substrate kit Cat#SK-4100, Vector Laboratories) for 5 min. Finally, Nissl staining using cresyl violet was performed.

### Histological analysis

Obtaining brightfield photomicrophotographs, image processing, and calculation of c-Fos^+^ cell density were performed as described elsewhere^21,33,57^. Brain regions abbreviations are based on Paxinos G. and Franklin K. B. J. mouse brain atlas^56^ with additional specifications of anterior (a) and posterior (p) parts. Due to ~ 60% success rate of the “trapped pup” rescue in virgin females, we counted c-Fos in both left and right hemispheres of “rescuer” and “non-rescuer” group brains (technical replicates) to increase the number of samples and reliability of results.

### Statistical analysis

All statistical analyses were conducted using R v. 4.4.0 (R Development Core Team, 2024; https://www.r-project.org/). No statistical methods were used to predetermine sample sizes, but simulation-based post-hoc power assessment was conducted for the first behavioral parameter of the current study (latency to pool entry) to illustrate power achieved in our behavioral paradigm (Supplementary Table S8).

For analyses of the latencies to the first pool entry and pool crossing (Figs. 1d, f), the numbers of pool entries and pool crossings (Figs. 1e, g), and the time spent in the pool **(**Fig. 1j) during the 5 min-long habituation test, as well as during the pup rescue test (Figs. 2b, c, d) and fist 5 min of both tests combined (Figs. 2f, g); for the analysis of multiple pup nurturing behaviors (Fig. 3a); for the analysis of latency to each pup nurturing behavior (Figs. 3b-g); for the analysis of pup care efficiency parameters (Figs. 3i, j) the generalized linear mixed model (GLMM) from glmmTMB package (v1.1.10) was used^58^. For analyses of the latencies to the first pool entry and pool crossing during the 5 min-long habituation test, as well as during the pup rescue test; for the analysis of latency to each pup nurturing behavior, Cox regression from the survival package (v 3.6.4; https://CRAN.R-project.org/package=survival) was used (Supplementary Figure S1). For analysis of correlation between latencies to pool entry and pool crossing, as well as between numbers of pool entry and pool crossing, Pearson correlation and Intraclass Correlation Coefficient (ICC) from the irr package (v0.84.1; https://cran.r-project.org/web/packages/irr/index.html) were used. For the analysis of density of c-Fos-positive neurons (Fig. 4c), Welch`s t-test and LMM from lme4 package (v1.1.35.3 )^59^ were used. Normality was assessed using the Kolmogorov–Smirnov test. GLMM tests were followed by Holm-adjusted *post-hoc* tests for comparisons between mouse groups and by Tukey-adjusted *post-hoc* tests for comparisons between water depth trials according to GraphPad Prism 10 Statistics Guide (https://www.graphpad.com/guides/prism/latest/statistics/stat_options_tab_two-way_anova.htm). Detailed information about statistical tests is provided in corresponding figure legend.

For the analysis of correlation between the latencies to rescue behaviors and densities of c-Fos-positive neurons in various brain regions (Fig. 4e), tydiverse (v2.0.0)^60^, ggdendro (v0.2.0; https://cran.r-project.org/web/packages/ggdendro/index.html), and corr (v0.4.4; https://cran.r-project.org/web/packages/corrr/index.html) packages were used. To create the heatmap demonstrating Spearman correlation results between c-Fos^+^ cell densities in examined brain regions and latencies to measured behaviors in the “trapped pup” rescue paradigm, each region’s individual value was first divided by the region’s average value for standardization. The pairwise Euclidean distances were then calculated between latencies to each behavior and between standardized c-Fos^+^ cell densities in each brain region. The computed distance matrix was used in the hierarchical clustering function (hclust) using Ward.D2 method for latencies and densities separately. In each step of clustering, the two clusters that lead to the smallest increase in the overall variance were merged. The resulting hierarchical clustering objects were then converted into dendrograms (as.dendrogram). Finally, the latency and density data were combined as a heatmap based on the results of Spearman correlation between each behavior latency and c-Fos^+^ cell density of each region. Behaviors that were displayed after the second tube opening were cut off due to their rarity and growing unreliability of the correlation results.

Error bars represent mean ± standard error of mean (S.E.M.). Statistical significance was indicated as follows: *p* < 0.0001 ****, 0.0001 ≤ *p* < 0.001 ***, 0.001 ≤ *p* < 0.01 **, 0.01 ≤ *p* < 0.05 *, 0.05 ≤ *p* < 0.1 †. Data from censored observations were replaced by the maximum observation time (1800 s (= 30 min) for all mice). The sample size is the same as the number of animals (biological replicate) in all cases, except for the c-Fos^+^ cell density data where technical replicates (left and right hemispheres) were utilized together with biological replicates.

## Supplementary Information

Below is the link to the electronic supplementary material.

Supplementary Material 1

Supplementary Material 2

Supplementary Material 3

Supplementary Material 4

## Data Availability

The datasets used and/or analyzed during the current study are available from the corresponding authors on reasonable request. Codes for behavioral analysis after DLC tracking and Spearman correlation matrix are available on GitHub (https://github.com/k-prokofeva/Prokofeva_Shibamiya_et_al_2026_code).
