# Supplementary material for: Neural correlates of adversity-overcoming pup rescue behavior in female mice
Source: Sci Rep. 2026 Mar 3;16:11844. doi: 10.1038/s41598-026-35639-7 (PMC13066482; doi:10.1038/s41598-026-35639-7)
Supplement: Supplementary file 1 — Supplementary Material 1 [file 41598_2026_35639_MOESM1_ESM.docx]

# **Neural correlates of adversity-overcoming pup rescue behavior in female mice**

# **Authors and affiliations:**

Kseniia Prokofeva^1,2*^, Mizuki Shibamiya^1, 2^, Rin Kawata^1^, Chihiro Yoshihara^1^, Kumi O. Kuroda^1,3*^

^1^ School of Life Science and Technology, Institute of Science Tokyo, Yokohama, Kanagawa 226-8501 Japan

^2^ These authors contributed equally: Kseniia Prokofeva and Mizuki Shibamiya

^3^ Laboratory for Circuit and Behavioral Physiology, RIKEN Center for Brain Science, Wako, Saitama 351-0198 Japan

# **Corresponding authors (email, full postal address):**

Kumi O. Kuroda

Email: [kurodalab@life.isct.ac.jp](mailto:kurodalab@life.isct.ac.jp)

School of Life Science and Technology, Institute of Science Tokyo, Yokohama, Kanagawa 226-8501 Japan

Kseniia Prokofeva

Email: [prokofeva.k.ab47@m.isct.ac.jp](mailto:prokofeva.k.ab47@m.isct.ac.jp)

School of Life Science and Technology, Institute of Science Tokyo, Yokohama, Kanagawa 226-8501 Japan


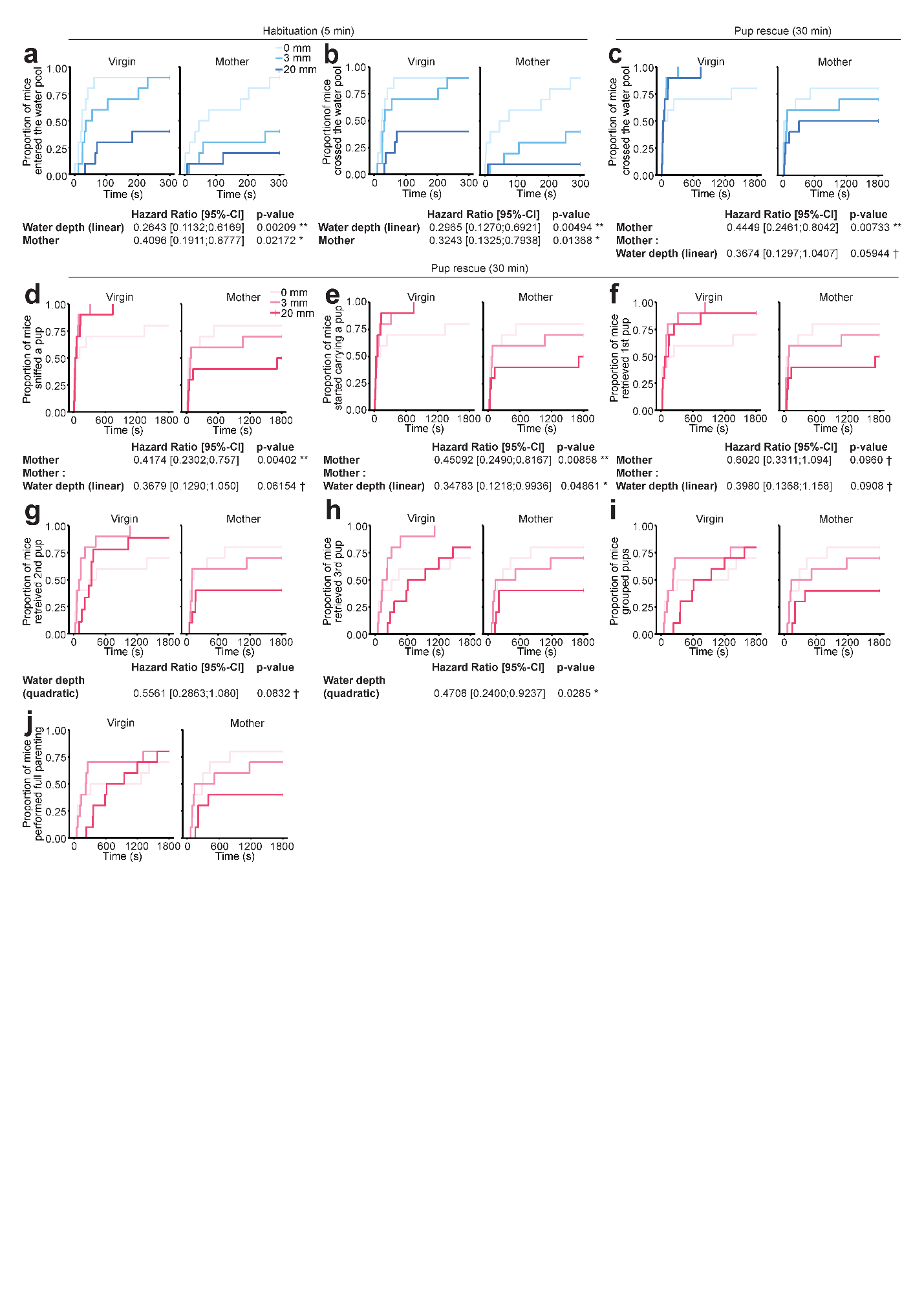
**Supplementary Figure S1. Analysis of aversion to water and pup care behaviors in adversity-overcoming rescue paradigm in female virgins and mothers using Cox regression.**

**a-c**, Proportion of mice that entered (**a**) or crossed (**b**) the water pool during habituation or pup rescue test (**c**). Cox regression (coxph(Surv(time, event) ~ mouse type * water depth) was used for analysis of this and following data.

**d-j**, Proportion of mice that reached pup sniffing (**d**), start of pup carrying (**e**), first pup retrieval (**f**), second pup retrieval (**g**), third pup retrieval (**h**), pup grouping (**i**), and full parenting (**j**) during the behavioral task in seconds. Due to rarity of full parenting behavior, statistical analysis was not applied to it. Data are shown as mean. Virgin mice, n = 10. Mother mice, n = 10. Effect size is reported in log-hazard scale.
